# Asymmetric lateral inhibitory neural activity in the auditory system: a magnetoencephalographic study

**DOI:** 10.1186/1471-2202-8-33

**Published:** 2007-05-17

**Authors:** Hidehiko Okamoto, Ryusuke Kakigi, Atsuko Gunji, Christo Pantev

**Affiliations:** 1 Department of Integrative Physiology, National Institute for Physiological Sciences, Myodaiji, Okazaki, Japan; 2 Institute for Biomagnetism and Biosignalanalysis, University of Muenster, Münster, Germany; 3 Department of Developmental Disorders, National Center of Neurology and Psychiatry, Tokyo, Japan

## Abstract

**Background:**

Decrements of auditory evoked responses elicited by repeatedly presented sounds with similar frequencies have been well investigated by means of electroencephalography and magnetoencephalography (MEG). However the possible inhibitory interactions between different neuronal populations remains poorly understood. In the present study, we investigated the effect of proceeding notch-filtered noises (NFNs) with different frequency spectra on a following test tone using MEG.

**Results:**

Three-second exposure to the NFNs resulted in significantly different N1m responses to a 1000 Hz test tone presented 500 ms after the offset of the NFNs. The NFN with a lower spectral edge closest to the test tone mostly decreased the N1m amplitude.

**Conclusion:**

The decrement of the N1m component after exposure to the NFNs could be explained partly in terms of lateral inhibition. The results demonstrated that the amplitude of the N1m was more effectively influenced by inhibitory lateral connections originating from neurons corresponding to lower rather than higher frequencies. We interpret this effect of asymmetric lateral inhibition in the auditory system as an important contribution to reduce the asymmetric neural activity profiles originating from the cochlea.

## Background

The amplitude and latency of the N1m response are known to depend on inter-stimulus intervals and frequency characteristics of the preceding sound [1-5]. Some early electroencephalographic experiments observed the strongest decrement of the N1 response, when the test stimuli and the intervening tones had equal frequencies [6,7]. This decrement has been mainly argued in terms of habituation and/or refractoriness [8-11]. However, both concepts mainly focus on the interaction of neural groups that are repeatedly activated by similar sound frequencies, but they did not consider the possible inhibitory interactions between different neural populations.

Neural activity from different receptive fields might also play an important role in the observed evoked response. Von Békésy [12] applied the lateral inhibition concept derived from other modalities to the auditory system. Not only the excitatory neural connections compose the afferent pathway, but also the inhibitory networks play an important role in the auditory afferent pathway. Lateral inhibition in the auditory system seems to contribute to the improvement of the perceptual contrast by enhancing the spectral edge of sound stimuli. A magnetoencephalography (MEG) study demonstrated that auditory evoked fields (AEFs) elicited by a band-passed noise centered at 1000 Hz frequency decreased after listening for several hours to music that had a spectral notch at around 1000 Hz [13]. The obtained decrement was considered to be the result of reversible functional deafferentation caused by long lasting lateral inhibition. In a further study, we compared the short-term effect of lateral inhibition and habituation in the auditory cortex [14]. For that purpose, we used a special noise, called comb-filtered noise (CFN), as a masker. The CFN was derived from white noise by applying multiple band-pass filters. The N1m responses elicited by two test stimuli before and after the exposure to the 3 s CFN were compared. One of the test stimuli was composed of the frequencies that overlapped with the frequencies of the CFN and thus in this case habituation would play an important role in the N1m decrements. The other stimulus was composed of the frequencies different from the frequencies in the CFN. In this case mainly the lateral inhibition effect would cause N1m decrements. As shown in this study, the three seconds exposure to the CFN caused significantly larger N1m decrement for the test stimulus that had no overlapping frequencies with the CFN. Therefore, it was concluded that the lateral inhibition effect might be stronger than the habituation effect in the human auditory cortex. The influence of the stop-bandwidths, which were eliminated by digital filtering from the white noise, was investigated in a follow-up study [15]. This study demonstrated the strongest lateral inhibition effect for a bandwidth of 1/4 octave, suggesting that the lateral inhibition might predominantly affect a certain range of frequency.

**Figure 1:**
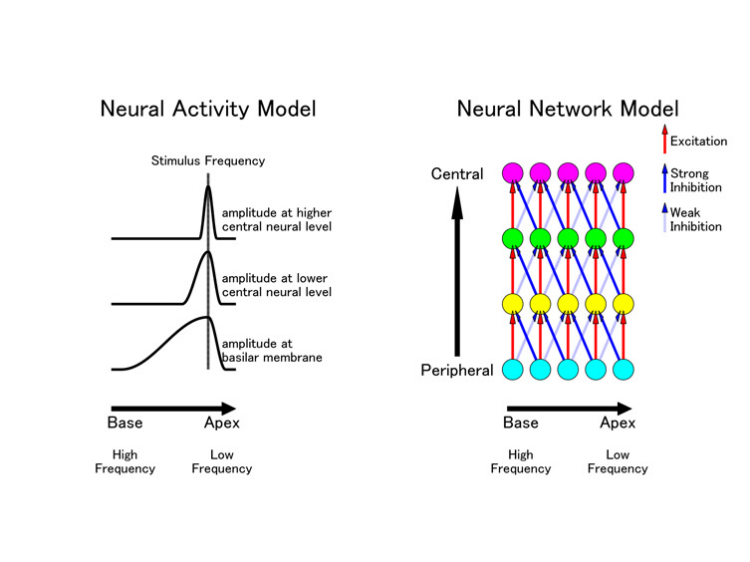
**Hypothesized neural network in auditory system**. Left: schematic diagram of hypothesized neural activity corresponding to a stimulus frequency from the peripheral to the central auditory pathway. Neural activity becomes sharper in the more central levels, especially in the high frequency range. Right: hypothetical excitatory and inhibitory neural network from the peripheral to the central auditory pathway. Red lines indicate excitatory neural connections and blue lines indicate inhibitory connections. Solid blue lines projecting from lower to higher frequencies have stronger inhibitory effects than the dashed blue lines projecting from higher to lower frequencies.

However, no study has yet differentiated the lateral inhibitory effect of neural connections on the following test stimulus from the lower and the higher spectral edges of the preceding masker. As a matter of fact the lateral inhibitory effect might be asymmetric in the auditory pathway because the auditory peripheral organ, the cochlea, has an asymmetric anatomical feature. The sound input causes the displacement of the basilar membrane in the cochlea. High frequency sounds activate only the neurons connected to the basal part of the cochlea, whereas low frequency sounds travel through the basal to the apical part. The amplitudes of displacement of the basilar membrane elicited by a low frequency sound increase gradually up to the maximum corresponding to the stimulus frequency and then drop steeply [16]. This asymmetric basilar membrane displacement, elicited by a low frequency sound, results in a shallow slope of the neural activities at the basal side (high frequency) compared to a steeper slope at the apical side (low frequency) of the cochlea (figure 1a). This asymmetric neural activity originating in the cochlea leads to asymmetrical frequency-tuning curves of the auditory nerve fibers [17] and inferior colliculus [18] with shallow tails in the lower frequencies, suggesting that low frequency sound with moderate intensity can activate neurons corresponding to high frequency sounds. However, the tail of the frequency-tuning curve becomes less evident in the central auditory neurons [19,20]. Thus, we could hypothesize that the stronger lateral inhibition from lower to higher frequency might contribute to the symmetrical frequency-tuning curve in the central auditory system (figure 1b). This assumption implies that the central auditory system might be able to compensate the asymmetric neural activity profiles originating in the cochlea by enhancing spectral contrasts, especially in frequency domains higher than the actual frequency of the applied auditory stimulus.

**Figure 2:**
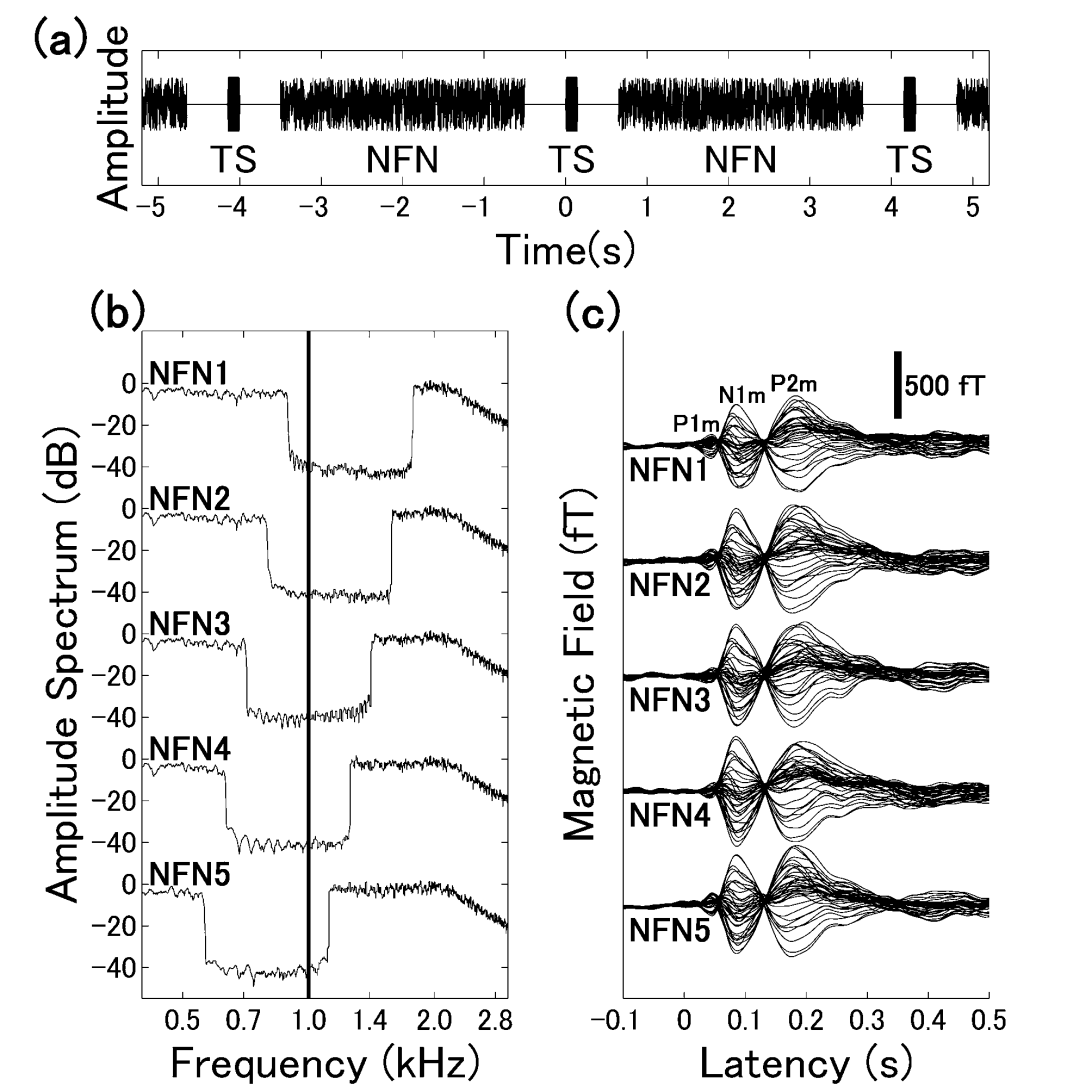
**Experimental design and representative single subject results**. (a): Schematic representation of the stimulus sequence with notch-filtered noise (NFN) of 3 s duration and the following test stimulus (TS) of 150 ms duration with an inter-stimulus interval of 500 ms. (b): Amplitude spectrum of the 3 s NFN measured at the ear-piece. Differences in frequency domain between the TS and the low-pass slope of the NFNs are: 1/6 octave (NFN1), 2/6 octave (NFN2), 3/6 octave (NFN3), 4/6 octave (NFN4), and 5/6 octave (NFN5). Center frequencies of the stop-band regions are: 1260 (NFN1), 1122 (NFN2), 1000 (NFN3), 891 (NFN4), and 794 Hz (NFN5). All NFNs had one-octave stop-band frequency. The bandwidths of the neighbouring dotted vertical lines are 1/6 octave. (c): Superposition of the auditory evoked magnetic fields (AEFs) from all magnetic sensors as recorded in one representative subject. AEFs elicited by the test stimulus (TS) following each NFN (NFN1 to NFN5).

To elaborate this hypothesis a masker with stop-band section, wide enough with respect to the lower and to the higher spectral edges, is needed to differentiate the inhibitory effects of those spectral edges. However, too wide a stop-band section might fail to elicit the lateral inhibitory effect. Considering these factors, we measured AEFs elicited by a test stimulus (TS) after exposure to maskers with one-octave bandwidth and center frequencies differing in 1/6-octave steps (figure 2a,b). The goal of this study was to investigate the lateral inhibitory effects of the lower and higher spectral edges of notch-filtered noises (NFNs) on the AEF response to a subsequent TS.

## Results

Clearly identifiable AEFs were obtained from all subjects. Figure 2c shows an example of individual magnetic field waveforms elicited by the TS for each condition. Because the TS were always the same pure tones of 1000 Hz, the corresponding responses showed small variances between conditions with clear N1m and P2m components peaking at approximately 100 and 180 ms after the stimulus-onset. However, in the present study we concentrated on the N1m component because of the uncertainty of the generator sites of the P2m [21], and because of the fact that the offset response to the 150 ms long TS might partly overlap with the P2m response [22]. Indeed, the calculated equivalent current dipoles (ECDs) for P2m had much lower goodness of fit than the ECDs for N1m; and in addition not all subject showed clear P1m and P2m responses.

The N1m is considered to have sources on Heschl's gyrus and the Planum Temporale [23]. In the present study we were able to explain more than 95% of the N1m field variance by one dipolar source in each hemisphere, a fact suggesting very good approximation of the N1m cortical sources. The N1m dipole source locations and orientations elicited by TS in the y-x plane (medial-lateral, posterior-anterior directions) and the y-z plane (medial-lateral, inferior-superior directions) showed no significant differences between conditions. Also, no significant difference was found for the latency between the five different conditions in either hemisphere.

**Figure 3:**
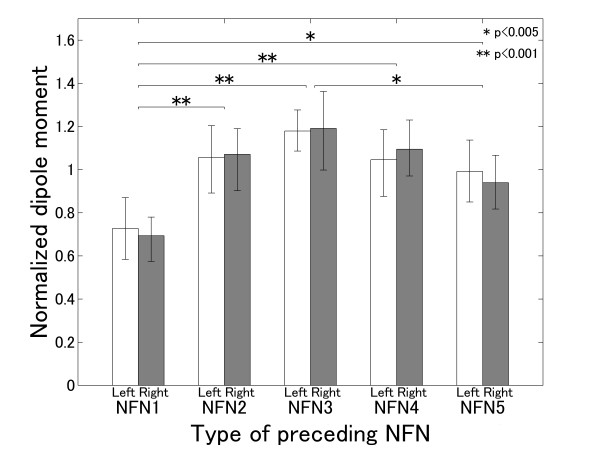
**Normalized N1m source strengths**. Group means (n = 9) of the normalized N1m source strength obtained by the test stimulus (TS) following each NFN (NFN1 to NFN5). Error-bars denote the 95 % confidence interval limits for the group means. White and gray bars represent responses from the left and right hemispheres, respectively.

The averages of the normalized N1m source strengths elicited by TS with their 95 % confidence interval limits are presented in figure 3. Repeated-measures analysis of variance (ANOVA) applied to the normalized N1m source strengths showed a highly significant effect of NFN-TYPE (F_(4,32) _= 12.4, *p *< 0.001). However, there was neither a significant effect of HEMISPHERE nor a significant interaction. The results displayed in figure 3 indicate that the smallest N1m response occurred after exposure to NFN1 and the largest after exposure to NFN3 in both hemispheres. As compared to NFN3 the N1m response is also smaller for NFN4 and NFN5. However, the decrease is not symmetric and it is more pronounced for the lower as compared to the higher frequency spectral edge. Post-hoc comparisons showed highly significant differences between NFN1 and NFN2 (*p *< 0.001), NFN1 and NFN3 (*p *< 0.001), NFN1 and NFN4 (*p *< 0.001), NFN1 and NFN5 (*p *< 0.005), and NFN3 and NFN5 (*p *< 0.005).

The N1m source locations and orientations elicited by the NFN-onsets were also analyzed, but they showed no significant differences between conditions. The corresponding normalized N1m source strengths and latencies elicited by the NFN-onsets also did not show any significant differences.

## Discussion

We observed how a decrease of N1m source strength, taken to be a reflection of lateral inhibition in the auditory pathway, depended in part on the frequency spectrum of the preceding NFN masker. Our previous study indicated that N1m decrement was dependent on the bandwidth of stop-band frequency of the preceding masker [15]. The present results demonstrated that lateral inhibitory effects from the lower and the higher spectral edges of NFN might be different along the auditory pathway. The most pronounced N1m decrement was obtained for the NFN1, which had a lower spectral edge of just 1/6 octave below the frequency of the test stimulus. Therefore, it can be assumed that the low frequency section of the NFN1 was the most influential on the N1m decrement. Similarly it could be assumed in case of NFN5 that the higher spectral edge of just 1/6 octave greater than the frequency of the test stimulus was mainly responsible for the N1m decrement. The N1m response elicited after exposure to NFN1 was significantly smaller than the one elicited after exposure to NFN5 even though both were equally distant from the test stimulus frequency. This result implies that the lower spectral edge of the NFN caused a larger N1m decrement than the higher spectral edge. The estimated source locations for N1m responses elicited by the TS with various preceding NFNs were not significantly different, a result suggesting that the neuronal group activated by the TS in NFN conditions did not differ, but rather the number of activated neurons and/or their level of synchrony did.

In this study, the TS and the stimulus timings remained constant in all conditions and only the preceding NFNs differed between the various conditions. Thus, only the type of the preceding NFN should be responsible for the difference in the N1m response elicited by the following TS. Of course, the preceding N1m response elicited by NFN-onset might also affect the N1m response elicited by the following TS-onset. However, the N1m responses to NFNs did not significantly differ in their source locations, source strengths, and latencies. Thus, we could hypothesize that the 3 s exposures to the NFN altered the responsiveness of the auditory neurons activated by the subsequent TS possibly via lateral connections, thus resulting in various N1m decrements.

The concept of lateral inhibition is similar to those suggested for other sensory systems showing excitatory and inhibitory interactions along the corresponding sensory pathway (c.f. figure 1b). Inhibitory lateral connections are a common model for contrast enhancement in sensory neural networks. In case of the auditory system, lateral inhibition seems to enhance spectral contrasts of sound inputs into the topographical frequency map, where neurons are systematically located with respect to specific frequency-tuning curves that exhibit a minimum threshold at a characteristic frequency (CF). Neurons with CFs outside the notch and close to the frequency slope of the NFN might receive less lateral inhibitory input from neighboring neurons with CFs inside the notch, since the latter are not excited by the NFN. This would result in an increased activation of the neurons with CFs around the edge frequency outside the notch. In contrast, neurons within the notch region are not excited, but still get strong inhibitory input via lateral connections from the neighboring neurons outside the notch, since the neighboring neurons are excited by the NFN. That results in a strongly inhibited activation of the neurons within the notch. Our previous study [14] has shown that the N1m responses elicited by the neural group corresponding to the spectral notch frequencies of CFN were more strongly decreased than the N1m responses corresponding to the pass-band frequencies after the 3 s CFN exposure.

We could ask the question if the asymmetric N1m decrements might be explained by another mechanism, e.g. habituation. The displacement of the basilar membrane has a shallow tail in the base area corresponding to higher frequencies (figure 1a). Thus, low frequency sounds could activate and habituate neurons with higher CFs more easily than high frequency sounds activate neurons with lower CFs. In the present study, the lower spectral edge of the NFN may cause a stronger habituation effect on auditory neurons corresponding to the TS frequency than the higher spectral edge. However, previous electroencephalographic studies showed that the population level habituation effect on N1 decrements was symmetrical [6,7]. These authors presented a test tone together with intervening tones with similar and different frequencies. The N1 decrement was maximal when the intervening tones were identical with the test stimulus, and the N1 became symmetrically larger as a function of the frequency difference between test tone and the intervening tone with higher or lower frequency. Therefore habituation alone could not explain the asymmetric N1m decrements observed in the present study.

Previous psychoacoustical study has shown that the effect of forward masking disappeared within 200 ms [24]. In this study as well as in previous ones [14,15] we have observed lateral inhibitory effects after the 500 ms silent interval between NFN and TS (figure 2a). Hence our results do not directly reflect the psychoacoustical forward masking effect. Also neurophysiological evidences obtained in animal studies indicated that the duration of the forward masking effect was less than 500 ms in the cochlea [25], auditory nerve [26,27], cochlear nucleus [28], and primary auditory cortex [29]. Mechanical lateral suppression caused by overlapping basilar membrane displacements and forward masking effects within the brainstem could not explain our results. Therefore we assume that the long lasting lateral inhibitory effect might be caused by inter-cortical inhibitory neural connections in the central auditory system [30,31]. In summary, the asymmetric N1m decrements observed in the present study imply that the lateral inhibition from lower to higher frequencies might have stronger effect than the one from higher to lower frequencies in cortical structures such as lateral aspects of the Heschl's gyrus and the temporal plane, which are known to be the cortical generator sites of N1m [32,33].

## Conclusion

The present study suggests that the effects of lateral inhibition might be asymmetric at the cortical level. Lateral inhibition from low to high frequency seems to be stronger compared to the one from high to low frequency. The asymmetric anatomical architecture of the basilar membrane results in asymmetrical auditory nerve activities with respect to frequency of a test sound. However, frequency-tuning curves become less asymmetric in higher stages of the auditory pathway. Therefore, we propose that asymmetric lateral inhibition in the central auditory system contributes to adjust the asymmetric neural activities originating in the cochlea. This adjustment results in sharper frequency-tuning and better auditory performance.

## Methods

### Subjects

Nine healthy right-handed subjects (three females, mean ± S.D. 29.5 ± 3.0 years) with no history of otological or neurological disorders participated in this study. Their hearing thresholds were normal in the frequency range from 250 to 8000 Hz as tested by means of pure tone audiometry (AA-71, Rion Co. Ltd., Japan) in a sound proof room. Subjects gave their informed consent to participate in the study, which was conducted in accordance with the Declaration of Helsinki and approved by the Ethics Committee of the National Institute for Physiological Sciences, Okazaki, Japan.

### Experimental design and stimulation

The design of the auditory stimulation paradigm is displayed in figure 2a. A test stimulus (TS) and an interfering NFN were successively presented. The stimulus onset asynchrony between two TS was 4.15 s. The inter-stimulus interval between any two sounds was 500 ms. The TS was always a 1000 Hz pure tone with duration of 150 ms. This timing was kept constant over the whole experiment and only the NFNs with duration of 3 s differed in their frequency spectra between conditions. Both TS and NFNs had 10 ms rise and fall time in order to avoid the perception of a click at the beginning and end of the sound. The NFNs were obtained from digitally filtered white noise using cutoff slopes greater than 100 dB/octave (Cool Edit 2000 sound editor, Syntrillium Software Corp., Arizona, United States). Amplitude spectrums of the 3 s NFNs measured at the ear-piece are displayed in the figure 2b. As shown in figure 2b there were no distortions around 1000 Hz caused by the audio-transferring system. The width of the stop-band of each NFN was one octave but with different center frequencies of 1260 Hz (NFN1), 1122 Hz (NFN2), 1000 Hz (NFN3), 891 Hz (NFN4), and 794 Hz (NFN5). Thus, the differences in the frequency domain between the pure tone TS at 1000 Hz and the lower spectral edge of each NFN were 1/6 octave (NFN1), 2/6 octave (NFN2), 3/6 octave (NFN3), 4/6 octave (NFN4), and 5/6 octave (NFN5). All stimuli were prepared as sound files and were presented through plastic tubes of 1.5 m length and silicon earpieces fitted to the subject's ears. The acoustic spectra (figure 2b) reflect the low-pass transfer characteristic of the sound transmission system above 2000 Hz. Both the TS and NFN were presented binaurally at an intensity of 45dB above individual sensation levels, which were determined at the beginning of each MEG session. In each session, 200TS trials for each NFN condition were presented in a randomized order.

### Data acquisition

AEFs were measured with dual 37-channel magnetometers (Magnes, Biomagnetic Technologies Inc., Ca, U.S.A.) centered over the C3 and C4 positions of the international 10–20 system for electroencephalographic placements in order to cover the auditory cortices of both hemispheres. The magnetic field signals were band-pass filtered between 0.1 to 200 Hz before sampling at a rate of 520.8 Hz. In order to keep subjects in an alert state and divert their attentional focus away from auditory stimuli, they watched a silent movie of their choice during the MEG recordings.

### Data analysis

Epochs of 600 ms magnetic field data, including 100 ms pre-stimulus interval, were selectively averaged for each experimental condition after rejection of epochs containing field changes larger than 3pT. The averaged magnetic field signals were then 30-Hz low-pass filtered and the DC offset was corrected based on the pre-stimulus interval. The source strength and position of a single equivalent current dipole (ECD) for N1m in each hemisphere was approximated at the latency around 100 ms after the TS-onsets. The origins of dipole locations and orientations were determined at the midpoint of the medial-lateral axis (y-axis) between the pre-auricular points of both ears. The posterior-anterior axis (x-axis) ran between the nasion and the origin, and the inferior-superior axis (z-axis) ran through the origin perpendicularly to the (x-y-plane). Estimates of the cortical source parameters were accepted for further evaluation only if the goodness of fit was above 95 %. The maximal source strengths of the N1m responses elicited by TS were normalized with respect to the average of the maximal N1m source strengths of all NFN conditions in each subject. A repeated-measures ANOVA with the normalized N1m source strengths as dependent variable and two within group factors (NFN-TYPE: NFN1, NFN2, NFN3, NFN4, and NFN5; HEMISPHERE: Left and Right) was calculated followed by post-hoc comparisons using Bonferroni-Dunn's correction with a significance threshold of *p *< 0.005. The N1m responses elicited by the NFN-onsets were also analyzed in similar way.

## Authors' contributions

HO conceived of the study, designed the experimental setup and the auditory stimuli. HO and AG acquired the data. HO performed the data & statistical analyses. All authors participated the data evaluation and interpretation and in writing the manuscript, and have approved the final version of the manuscript.
